# GOLPH3 promotes endotoxemia-induced liver and kidney injury through Golgi stress-mediated apoptosis and inflammatory response

**DOI:** 10.1038/s41419-023-05975-x

**Published:** 2023-07-21

**Authors:** Theodomir Dusabimana, Jihyun Je, Seung Pil Yun, Hye Jung Kim, Hwajin Kim, Sang Won Park

**Affiliations:** 1 Department of Pharmacology, Institute of Health Sciences, Gyeongsang National University College of Medicine, Jinju, 52727 Republic of Korea; 2 Anti-aging Bio Cell factory Regional Leading Research Center (ABC-RLRC), Gyeongsang National University, Jinju, 52727 Republic of Korea; 3 Department of Convergence Medical Sciences, Gyeongsang National University Graduate School, Jinju, 52727 Republic of Korea

**Keywords:** Sepsis, Golgi

## Abstract

Sepsis is a serious clinical condition characterized by a systemic inflammatory response, a leading cause of acute liver and kidney injury, and is associated with a high morbidity and mortality. Understanding the molecular mechanisms underlying the acute liver and kidney injury is crucial for developing an effective therapy. Golgi apparatus plays important roles and has various substrates mediating cellular stress responses. Golgi phosphoprotein 3 (GOLPH3), linking Golgi membranes to the cytoskeleton, has been identified as an important oncogenic regulator; however, its role in endotoxemia-induced acute liver and kidney injury remains elusive. Here, we found that upregulation of GOLPH3 was associated with endotoxemia-induced acute liver and kidney injury. Lipopolysaccharide (LPS) treatment increased Golgi stress and fragmentation, and associated pro-inflammatory mediator (*Tnfα, IL-6*, and *IL-1β)* production in vivo and in vitro. Interestingly, the downregulation of GOLPH3 significantly decreased LPS-induced Golgi stress and pro-inflammatory mediators (*Tnfα, IL-6, Mcp1*, and *Nos2)*, and reversed apoptotic cell deaths in LPS-treated hepatocytes and renal tubular cells. GOLPH3 knockdown also reduced inflammatory response in LPS-treated macrophages. The AKT/NF-kB signaling pathway was suppressed in GOLPH3 knockdown, which may be associated with a reduction of inflammatory response and apoptosis and the recovery of Golgi morphology and function. Taken together, GOLPH3 plays a crucial role in the development and progression of acute liver and kidney injury by promoting Golgi stress and increasing inflammatory response and apoptosis, suggesting GOLPH3 as a potential therapeutic target for endotoxemia-induced tissue injury.

## Introduction

Sepsis is a life-threatening clinical condition with systemic inflammatory responses triggered by a dysregulated host response to infection, leading to progressive multi-organ failure [1, 2]. The global burden of sepsis accounts 48.9 millions of infected cases and 11 millions of sepsis related-deaths in 2017, representing a mortality rate of 19.7% [3]. A pathogen-induced systemic inflammation and subsequent immunosuppression are key features responsible for a high mortality rate of sepsis, which is still incurable with current therapeutic strategies such as broad-spectrum antibiotic therapy or fluid resuscitation [4]. Therefore, there is a need for developing new therapeutic interventions for treating sepsis.

Systemic activation of immune system by pathogen-associated molecular patterns (PAMPs) and damage-associated molecular patterns (DAMPs) in sepsis induces severe and persistent inflammatory responses characterized by an excessive of inflammatory cytokines and chemokines, termed as “cytokine storm”. Classically, the mortality distribution occurs in a biphasic pattern, initially due to inadequate resuscitation and cardiac and pulmonary failure, followed by organ failure and immune dysfunction. The microvascular alterations and endothelial dysfunction during sepsis also contribute to a complex systematic response and multiple organ dysfunction. Recently, a trimodal distribution is prominent with reduced magnitude in the two early peaks and a third uprising peak 2–3 months after sepsis [5]. The delayed sepsis mortality in the early stages is attributed to advanced care of elderly and complex patients in intensive care units. Innate immune dysfunction and adaptive immune suppression together facilitate multiple organ injury and persistent infectious states, resulting in patient death. A progression to severe sepsis and septic shock is associated with profound circulatory, cellular, and metabolic abnormalities, greatly increasing a risk of mortality [6]. Liver and kidney are often the major organs to be affected and damaged by sepsis, due to their critical role in hemodynamic and cellular homeostasis, subsequently leading to acute liver injury (ALI) or acute kidney injury (AKI); therefore, therapeutic approaches aimed at protecting these organs in the early stages of sepsis significantly reduce mortality [7, 8].

The liver plays an important role in many physiological and pathological processes including immune response, detoxification, metabolism and cellular homeostasis, which makes this organ extremely vulnerable to the circulating antigens, danger signals, and endotoxins such as lipopolysaccharide (LPS) after microbial infection [9, 10]. The hepatic defensive mechanisms of immune activation in sepsis patients are important for clearing microbial products; however, excessive systemic inflammatory response induces hepatocellular damage and organ failure [6, 10]. Thus, balancing immune activation and suppression and understanding the underlying molecular mechanisms in the pathogenesis of ALI may help to decipher new therapeutic strategies for ameliorating sepsis-induced ALI.

The endotoxemia-induced kidney injury is associated with prolonged inflammatory responses, oxidative stress, tubular cell death, and metabolic dysfunction, ultimately leading to kidney failure. [9, 11, 12]. These abnormalities are major features for initiating AKI, and critical risk factors for developing chronic kidney disease (CKD) [11, 13]. AKI occurs in 20–50% of sepsis patients in intensive care units, and is accompanied with multiple organ dysfunction, resulting in a higher mortality rate than in patients with sepsis alone [8, 14, 15]. The pathogenesis of sepsis-induced AKI is complex and the underlying mechanisms are under active investigation. Thus, there is an emergent need for identifying the mechanisms and novel therapeutic agents for sepsis-induced AKI.

The Golgi apparatus is an important cytoplasmic organelle which involves in post-translational modification, trafficking and sorting of both lipids and proteins synthesized from endothelium reticulum (ER), distributing them to appropriate cellular locations via endocytosis and exocytosis [16, 17]. In mammals, Golgi apparatus is often localized to the perinuclear region of the cells, with a highly organized and ribbon-like structure, composed of elongated cisternae stacks [16, 17]. Increasing evidence suggests that the Golgi apparatus is a sensor and common downstream effector of stress signals in cell death pathways [18, 19]. Disruption of Golgi structure and functions, termed as Golgi stress, initiates Golgi disassembly and fragmentation, and has been reported in various diseases including neurodegenerative disorders, stroke, alcohol-related liver diseases, viral hepatitis, hepatocellular or renal carcinoma and other type of cancers [16, 18–23]. However, the molecular basis for Golgi stress and its role in the disease progression remain largely unknown.

Golgi phosphoprotein 3 (GOLPH3) is located at Golgi membrane, the cytoplasmic face of trans-Golgi network and on the vesicles secreted by Golgi apparatus. GOLPH3 exhibits diverse cellular functions such as maintenance of Golgi structure, secretory and trafficking pathways, regulation of organelle crosstalk, and related signaling pathways [18, 24]. GOLPH3 pathway plays a critical role in anterograde trafficking to the plasma membrane. GOLPH3 directly interacts with phosphatidylinositol 4-phosphate (PtdIns4P), and tightly binds to unconventional myosin 18A (MYO18A) to create a tensile force required for vesicle budding and anterograde trafficking and maintenance of the Golgi morphology [25, 26]. GOLPH3 has been identified as a novel oncogene protein that plays a key role in cell proliferation and tumor progression by regulating phosphatidylinositol 3-kinase (PI3K)-protein kinase B (PKB, also known as AKT) and mammalian target of rapamycin (mTOR) signaling pathways [27]; GOLPH3 is upregulated in many solid human tumors [18, 24, 25, 27–29]. Recent studies have demonstrated that GOLPH3 expression is upregulated in oxygen-glucose deprivation and reoxygenation injury and endotoxemia-induced lung injury [30, 31]. GOLPH3 may act as a sensor of Golgi stress during oxidative stress or cytotoxic insults and propagates stress signals to the downstream effectors to alter cellular functions [18]. However, the pathogenic mechanisms of GOLPH3 and Golgi stress are still controversial. In this study, we investigated the role of GOLPH3 in the development and progression of endotoxemia-induced ALI and AKI.

## Materials and methods

### Experimental animals

Wild-type male C57BL/6 mice (8–10 weeks) were purchased from KOATECH Co. (Pyeongtaek, South Korea) and maintained in the animal facility at Gyeongsang National University. All animal experiments were approved by the Institutional Board of Research at Gyeongsang National University, and performed in accordance with the National Institute of Health (NIH) guidelines for laboratory animal care. Mice were housed under alternating 12 h light/dark cycles, at temperature of 22–24 °C with a relative humidity of 40–60% and provided with sterilized water and standard chow *ad libitum* (Harlan Laboratories, IN, USA).

### Animal model of endotoxemia-induced liver and kidney injury

Acute liver and kidney injury were induced in C57BL/6 mice by a single injection of bacterial endotoxin (LPS, 20 mg/kg) from *Escherichia coli* O111:B4 (Sigma-Aldrich, St. Louis, MO, USA). The mice were randomly divided into two groups; LPS and control groups of mice were intraperitoneally injected with LPS and saline, respectively (*n* = 10), and all mice were sacrificed at 18 h post injection. Blood was collected from an inferior vena cava using a heparinized syringe, and plasma was separated by centrifugation at 3000 × *g* for 15 min, and stored at −80 °C for biochemical analysis. Liver and kidney tissues were collected, snap-frozen in liquid nitrogen for storage at −80 °C or fixed in 10% buffered formalin for further analysis.

### Cell culture and treatment

Mouse hepatocytes (FL83B, #CRL-2390) were purchased from ATCC (Manassas, VA, USA) and cultured in Kaighn’s modification of Ham’s F-12 medium (F12K, Thermo Fisher Scientific, Waltham, MA, USA), supplemented with 10% fetal bovine serum (FBS) and 1% penicillin/streptomycin (HyClone Laboratories, Logan, UT, USA). The human proximal tubular epithelial cells (HK-2, #CRL-2190) were purchased from ATCC and maintained in Dulbecco’s modified Eagle medium (DMEM)/F12K medium (Thermo Fisher Scientific), supplemented with 10% FBS and 1% penicillin/streptomycin (HyClone Laboratories). RAW 264.7 macrophage cells were maintained in DMEM medium containing 4.5 g/l of glucose, supplemented with 10% FBS and 1% penicillin/streptomycin (HyClone Laboratories). Cells were maintained at 37 °C in a humidified incubator containing 5% CO_2_ and 95% air, and stimulated by LPS (1 µg/ml) to mimic an endotoxemia model in vitro. GOLPH3 specific small interfering RNA (siRNA) and corresponding control siRNA were purchased from Bioneer (Daejeon, South Korea). For transfection, the cells were transfected using the Lipofectamine^TM^ RNAiMax reagent (Thermo Fisher Scientific) by following the manufacturer’s instructions.

### Biochemical assays

Plasma alanine aminotransferase (ALT), aspartate aminotransferase (AST), and blood urea nitrogen (BUN) were measured using commercial assay kits from IVD Lab (Uiwang, South Korea) and a UV-180 spectrophotometer (Shimadzu, Tokyo, Japan). Plasma creatinine was measured by a direct colorimetric Jaffe method and detected using a spectrophotometer, as previously described [32]. Plasma TNFα and IL-6 levels were determined using enzyme-linked immunosorbent assay (ELISA) kits from Invitrogen (Waltham, MA, USA) according to the manufacturer’s instructions.

### Histological evaluation of acute liver and kidney injury

The 10% formalin-fixed liver and kidney tissues were embedded in paraffin and sectioned at 5 µm of thickness. The tissue sections were then stained with Hematoxylin and Eosin (H&E) according to a standard protocol and analyzed using a CKX41 light microscopy (Olympus, Tokyo, Japan). The severity of liver damage was examined, and histological liver injury scores for each sample were assessed as the sum of individual scores given for 3 different parameters based on the following Suzuki criteria: congestion (None = 0, Minimal =1, Mild = 2, Moderate = 3, Severe = 4), vacuolization (None = 0, Minimal = 1, Mild = 2, Moderate = 3, Severe = 4), and necrosis (None = 0, single cell necrosis = 1, < 30% = 2, 30–60% = 3, >60% = 4); scores for each parameter are ranged from 0 to 4, with a maximum score of 12, as previously described [33, 34]. To examine the severity of kidney damage, histological renal injury scores were assessed semi-quantitatively in randomly selected samples and graded as follows: Grade 0= normal, grade 1 = < 10%, grade 2 = 10–25%, grade 3 = 25–50%, grade 4 = 50–75%, and grade 5 = 75–100% of renal abnormalities including tubular cell necrosis, tubular atrophy or dilation, loss of brush border, vacuolization, tubular epithelial cell shedding, cast formation and segmental glomerular lesions [35, 36].

### Immunohistochemistry (IHC) analysis

The 10% formaldehyde-fixed, paraffin-embedded liver or kidney tissue sections were prepared. Briefly, the fixed tissue sections were deparaffinized, rehydrated, and antigen-retrieved in sodium citrate buffer (10 mM, pH 6.0) for 20 min. Subsequently, the sections were permeabilized with 0.3% Triton X-100 for 10 min in PBS. The sections were blocked using 10% normal horse serum and incubated with a primary antibody against GOLPH3 (Proteintech Group, Chicago, IL, USA) at 4 °C overnight. Next day, the sections were washed and incubated with a biotinylated secondary antibody (Vector Laboratories, Burlingame, CA, USA) for 1 h at room temperature. The sections were then incubated in the avidin-biotin-peroxidase complex solution (ABC solution; Vector Laboratories) for 30 min, and labeled by adding 3,3’-diaminobenzidine (DAB) Peroxidase Substrates (Vector Laboratories), being counterstained with Mayer’s Hematoxylin. Finally, the sections were mounted and examined using a CKX41 light microscope (Olympus). GOLPH3-positive cells were analyzed and counted using ImageJ software (NIH, Bethesda, MD, USA).

### Immunofluorescence (IF) staining

Formaldehyde-fixed, paraffin-embedded liver or kidney tissue sections were deparaffinized, rehydrated, antigen retrieved in sodium citrate buffer (10 mM, pH 6.0) for 20 min. Tissue sections or cells fixed in 4% paraformaldehyde were permeabilized with 0.3% Triton X-100 in PBS and blocked with 10% normal horse serum. The samples were then immune-stained with specific primary antibodies of interests (Golgi matrix protein of 130 kDa (GM130) and GOLPH3) at 4 °C overnight. After washing, the samples were subsequently incubated with a corresponding secondary antibody conjugated with Alexa Fluor® 488 or 594 for 1 h at room temperature and mounted with ProLong Gold Anti-fade mounting solution containing DAPI (Thermo Fisher Scientific). All samples were examined using Olympus Fluoview FV1000 confocal microscope (Olympus) and analyzed by using ImageJ software (NIH). For the Golgi morphology assessment, fragmented Golgi was defined as scattered dots (not connected) in the perinuclear region or multiple mini-golgi (isolated dots) dissociated from the major Golgi apparatus based on GM130 staining, a Golgi marker. Quantification of cells with fragmented Golgi per high power field (HPF) was performed using more than 300 cells per experiment with ImageJ software (NIH) as previous described [16, 17, 37].

### Terminal deoxynucleotidyl transferase dUTP nick-end labeling (TUNEL) assay

TUNEL staining was performed to evaluate the degree of apoptosis using an In Situ Cell Death Fluorescein Detection Kit (Roche Molecular Biochemicals, Mannheim, Germany) according to the manufacturer’s instructions and the images were captured using Olympus Fluoview FV1000 confocal microscopy. The number of TUNEL-positive cells/HPF was quantified from each group, using ImageJ software (NIH).

### Western blot analysis

Immunoblotting was performed as previously described [33, 38]. Briefly, tissue or cell lysates were prepared in ice-cold radio-immunoprecipitation assay (RIPA) buffer with protease inhibitors (Thermo Fisher Scientific), sonicated and incubated for 20 min, and the protein concentration was determined using a Pierce^TM^ BCA Protein Assay Kit (Thermo Fisher Scientific). The protein lysates were separated using SDS-PAGE and transferred to PVDF membranes and blocked with 5% skim milk or 3% bovine serum albumin (BSA). The membranes were incubated with primary antibodies against GOLPH3, Golgi reassembly-stacking protein 1 (GRASP65) and ADP-ribosylation factor 4 (ARF4) (Proteintech), phosphatidylinositol 4-kinase III beta (PI4KIIIβ) (Abcam, Cambridge, UK), MYO18A (Santa Cruz Biotechnology, Dallas, TX, USA), phosphorylated nuclear factor kappa B (p-NF-κB) p65, NF-κB p65, phosphorylated NF-κB inhibitory subunit alpha (p-IκBα), IκBα, p-AKT, AKT, inducible nitric oxide synthase (iNOS) and cyclooxygenase-2 (COX-2) (Cell Signaling Technology, Danvers, MA, USA) and β-actin (Sigma-Aldrich) in the blocking solution at 4 °C overnight. Next, the membranes were incubated with the appropriate horseradish peroxidase (HRP)-conjugated secondary antibodies (Bio-Rad, Hercules, CA, USA) at room temperature for 1 h and the antigen-antibody complex was detected using a ChemiDoc XRS+ System (Bio-Rad). Relative protein levels were quantified using Image Lab^TM^ software (Bio-Rad). The detailed information of antibodies is described in Supplementary Table 1.

### Quantitative real-time polymerase chain reaction (PCR) analysis

The total RNA was extracted with Trizol (Invitrogen, Carlsbad, CA, USA) and converted into cDNA using the RevertAid Reverse Transcription System (Thermo Fisher Scientific) according to the manufacturer’s protocol. Real-time PCR analysis was performed with a CFX Connect real-time PCR System by using iQ SYBR Green Supermix (Bio-Rad). Real-time PCR analysis was performed with initial denaturation at 94 °C for 5 min and the cycling conditions were 45 cycles of 10 s at 95 °C, 10 s at 60 °C, and 30 s at 72 °C. Relative mRNA levels were normalized to those of glyceraldehyde 3-phosphate dehydrogenase (GAPDH) for each gene. The primer sequences are listed in Supplementary Table 2.

### Statistical analysis

Statistical significance was determined using one-way analysis of variance (ANOVA), followed by Bonferroni’s multiple comparisons for multiple groups or unpaired two-tailed Student’s *t*-test to compare two groups (GraphPad Prism 7 Software, v.7.00, La Jolla, CA, USA). The data are expressed as the mean ± SEM. A *p*-value < 0.05 was considered statistically significant.

## Results

### GOLPH3 is upregulated in endotoxemia-induced acute liver and kidney injury

**Fig. 1:**
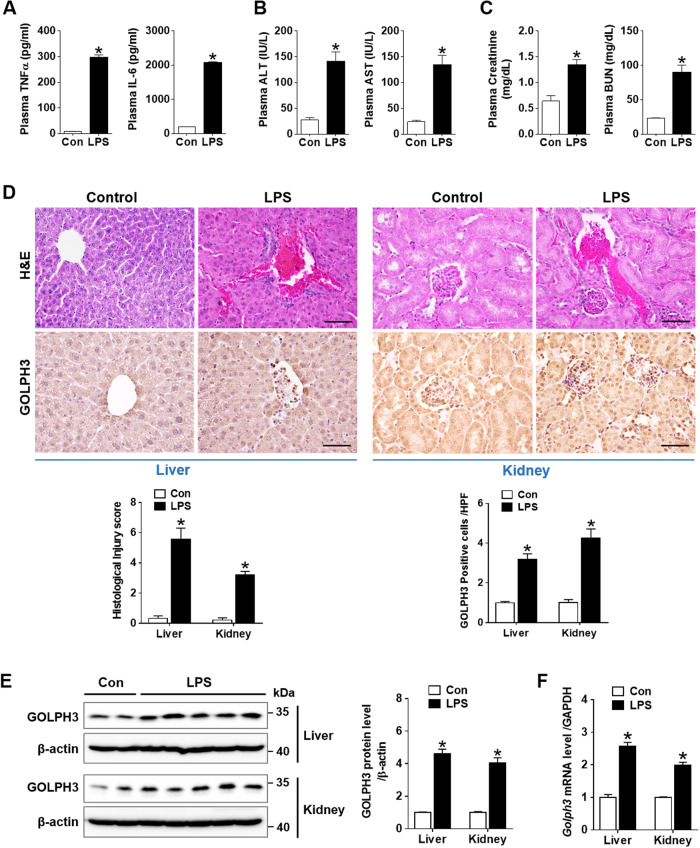
GOLPH3 is upregulated in endotoxemia-induced acute liver and kidney injury in mice. C57BL/6 mice were intraperitoneally injected with a single dose of LPS (20 mg/kg) or normal saline (control) and sacrificed at 18 h post injection. Blood, liver, and kidney tissue samples were collected at sacrifice. **A** Plasma TNFα and IL-6 cytokine production was determined by ELISA. **B**, **C** Plasma liver and kidney injury parameters (ALT, AST, Creatinine, and BUN) were measured respectively with a spectrophotometer (*n* = 4–6). **D** Representative images of histopathological changes are shown by H&E staining (upper panel) and GOLPH3 immunostaining (lower panel) in liver and kidney tissues. The extent of liver or kidney damage was evaluated and graded as described in the method section, while GOLPH3-positive cells/high power field (HPF) were assessed and counted (n = 3–5). Scale bar, 100 µm. **E** Western blot analysis and quantification of GOLPH3 protein levels over β-actin as a loading control (*n* = 3–5). **F** Relative mRNA levels of GOLPH3 were examined using real-time PCR analysis in liver and kidney tissues, and relative mRNA expression was normalized to that of GAPDH (*n* = 3–5). The data are presented as mean ± SEM. Two-tailed Student’s *t*-test was used, *p < 0.05 versus Control.

To investigate a potential role of GOLPH3 in the development and progression of endotoxemia-induced liver and kidney injury, an endotoxemia mouse model was generated by intraperitoneal injection of a single dose of LPS (20 mg/kg; Supplementary data 1A). Plasma TNFα and IL-6, key biomarkers of endotoxemic injury, were significantly increased in LPS-treated mice compared to control (Fig. 1A). Plasma ALT and AST for liver damage, and plasma creatinine and BUN for kidney injury, were significantly increased in LPS-treated mice compared to control (Fig. 1B, C). Histopathological changes were evaluated by H&E staining to further confirm liver and kidney damage (Fig. 1D, upper panel). GOLPH3 expression increased in liver and kidney tissues of LPS-treated mice (Fig. 1D, lower panel). LPS treatment significantly upregulated the expression of GOLPH3 protein and mRNA in multiple organs, including liver and kidney (Fig. 1E, F and Supplementary data 1B). These results strongly support that upregulation of GOLPH3 is associated with endotoxemia-induced liver and kidney dysfunction.

### LPS-induced endotoxemia promotes Golgi stress and inflammatory response in liver and kidney

**Fig. 2:**
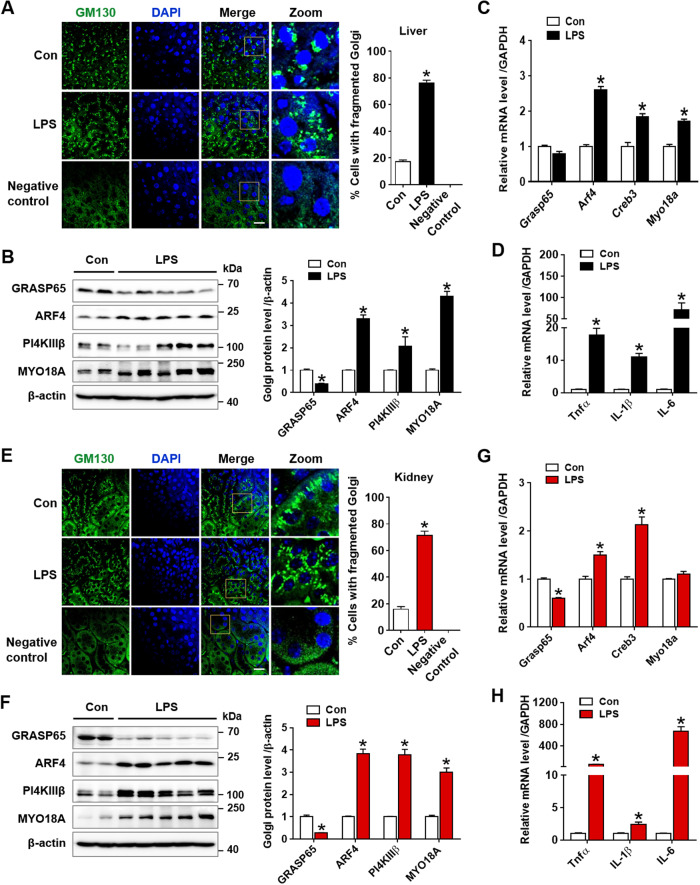
LPS-induced endotoxemia promotes Golgi stress and inflammatory response in acute liver and kidney injury. **A** Immunofluorescence staining of GM130 (green), a marker of Golgi morphology, in the liver tissues was assessed by confocal microscopy and quantification of cells with fragmented Golgi from each group is shown (*n* = 3). Scale bar, 20 µm. **B** Golgi functional and structural proteins (GRASP65, ARF4, P14KIIIβ, and MYO18A) were examined using western blot analysis in liver tissues and relative expression was shown over β-actin as loading control (*n* = 3–5). **C** Relative mRNA levels of *Grasp65, Arf4, Creb3*, and *Myo18a* were determined by real-time PCR analysis in liver tissues, and relative mRNA expression was normalized to that of GAPDH (*n* = 3–5). **D** Relative mRNA levels of pro-inflammatory cytokines (*Tnfα, IL-1β*, and *IL-6)* were determined by real-time PCR analysis in liver tissues and relative mRNA expression was normalized to that of GAPDH (*n* = 4). **E**–**H** As described above, the experiments in (**A**), (**B**), (**C**), (**D**) were similarly performed in kidney tissues. The data are presented as mean ± SEM. Two-tailed Student’s *t*-test was used, *p < 0.05 versus Control.

The Golgi apparatus plays an important role to sense and transduce stress signals through crosstalk with the ER and mitochondria in cell death pathways [18]. The Golgi structure, ribbon-like stack formation are coordinated by resident Golgi matrix proteins such as Golgi reassembly-stacking protein 1 and 2 (GRASP65 and GRASP55, respectively), and Golgins (GM130 and Golgin-45) [39, 40]. Disruption of its stacking morphology causes fragmentation and dysfunction of the Golgi apparatus, alters protein modifications, sorting, and trafficking processes, and leads to liver dysfunction and neurodegeneration [16, 17]. GOLPH3 binds to PI4KIIIβ and MYO18A, linking to the Golgi and actin cytoskeleton, which is required for an efficient Golgi-to-plasma membrane protein trafficking. Activation of the GOLPH3 pathway by overexpression of PI4KIIIβ or GOLPH3, or upon DNA damage, results in Golgi fragmentation and impaired trafficking [25]. In addition, the cAMP-responsive element binding protein 3 (CREB3) and ARF4 signaling pathway, is shown to mediate the Golgi stress response to pathogens [41]. To examine the Golgi stress and inflammatory response in the development of endotoxemia-induced liver and kidney damage, we first investigated Golgi integrity by immunofluorescence staining of GM130, a Golgi marker. LPS-treated mice exhibited an altered Golgi morphology with increased expansion and fragmentation compared to control mice, indicating Golgi stress and associated hepatic and renal injury (Fig. 2A, E). Next, we analyzed the expression of Golgi matrix proteins and found that LPS treatment decreased GRASP65 protein levels, and increased ARF4, PI4KIIIβ, and MYO18A protein levels in liver and kidney tissues (Fig. 2B, F). Consistently, real-time PCR analysis showed that LPS treatment increased mRNA levels of *Arf4, Creb3*, and *Myo18a*, but decreased *Grasp65* levels in the liver and kidney tissues (Fig. 2C, G). These changes were accompanied by the induction of pro-inflammatory mediators (*Tnfα, IL-1β*, and *IL-6*), possible downstream effectors of Golgi stress (Fig. 2D, H). LPS treatment increased pro-inflammatory cytokines in multiple organs including liver, kidney, lung, and intestine (Supplementary data 1C). These findings indicate that LPS-induced endotoxemia promotes Golgi stress and inflammatory response, which exacerbates liver and kidney injury.

### Induction of GOLPH3 is associated with Golgi stress and inflammatory response in vitro

**Fig. 3:**
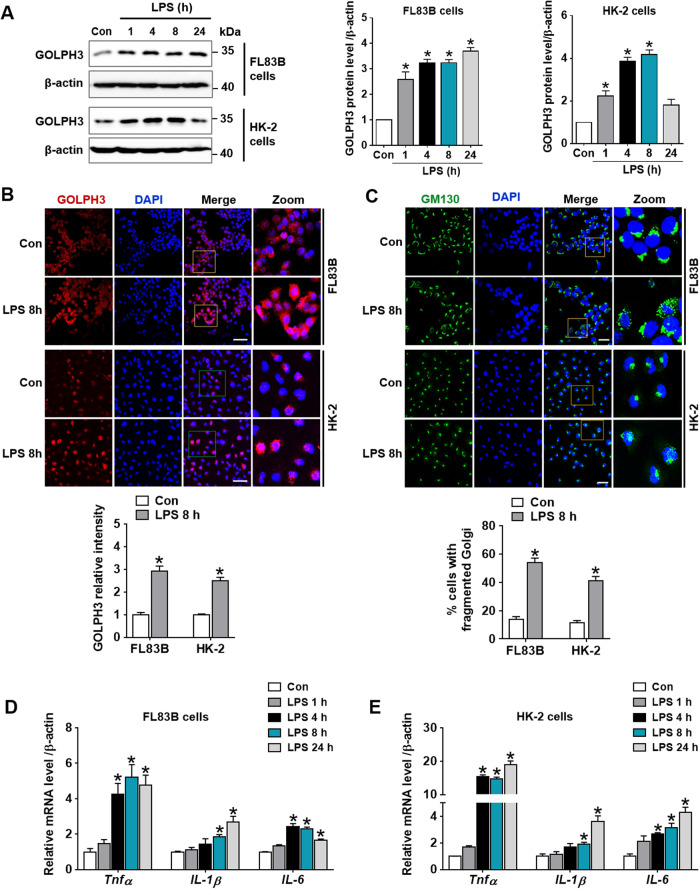
Upregulation of GOLPH3 by LPS treatment is associated with Golgi stress and inflammatory response in vitro. **A** Hepatocytes (FL83B) and renal proximal tubule cells (HK-2) were treated with LPS (1 µg/ml) for different time points as indicated. The protein expression levels of GOLPH3 were assessed by western blot analysis, and the quantification results are shown (*n* = 3). **B** The cells were treated with LPS (1 µg/ml) for 8 h, representative images of immunofluorescence staining of GOLPH3 (red) were analyzed by confocal microscopy and relative GOLPH3 intensity were quantified (*n* = 3). Scale bar, 50 µm. **C** Immunofluorescence staining of GM130 (green) was performed to examine the Golgi morphology. The representative immunofluorescence staining images of GM130 were examined using confocal microscopy. Relative cells with abnormal and fragmented Golgi were quantified (*n* = 3). Scale bar, 20 µm. **D**, **E** Relative mRNA levels of pro-inflammatory cytokines (*Tnfα, IL-1β*, and *IL-6*) were examined by real-time PCR analysis in FL83B and HK-2 cells, respectively; and relative mRNA expression of each gene was normalized to that of GAPDH (*n* = 3). The data are presented as mean ± SEM. One-way ANOVA, followed by Bonferroni’s multiple comparisons was used, *p < 0.05 versus Control.

As previously reported, GOLPH3 expression is upregulated in oxidative injury and various pathologies [18, 29, 30]. This study investigated whether GOLPH3 is associated with endotoxemia-induced hepatic and renal injury. Since the induction of GOLPH3 and Golgi fragmentation were found in the liver and kidney of LPS-treated mice, these changes were further analyzed in vitro using hepatocytes (FL83B) and proximal tubular epithelial cells (HK-2). The protein and mRNA levels of GOLPH3 were significantly increased in FL83B and HK-2 cells in a time-dependent manner after LPS treatment (Fig. 3A, Supplementary data 2A, B). Further, GOLPH3 expression and Golgi fragmentation were evaluated by immunofluorescence staining of GOLPH3 and GM130; GOLPH3 and Golgi fragmentation were upregulated in both cells after LPS treatment (Fig. 3B, C). LPS-treated cells increased pro-inflammatory cytokines (*Tnfα, IL-1β*, and *IL-6)*, which may be associated with GOLPH3 induction (Fig. 3D, E). These results suggest that upregulated GOLPH3 and Golgi fragmentation are associated with inflammatory response upon LPS treatment.

### Downregulation of GOLPH3 improves LPS-induced Golgi stress response

**Fig. 4:**
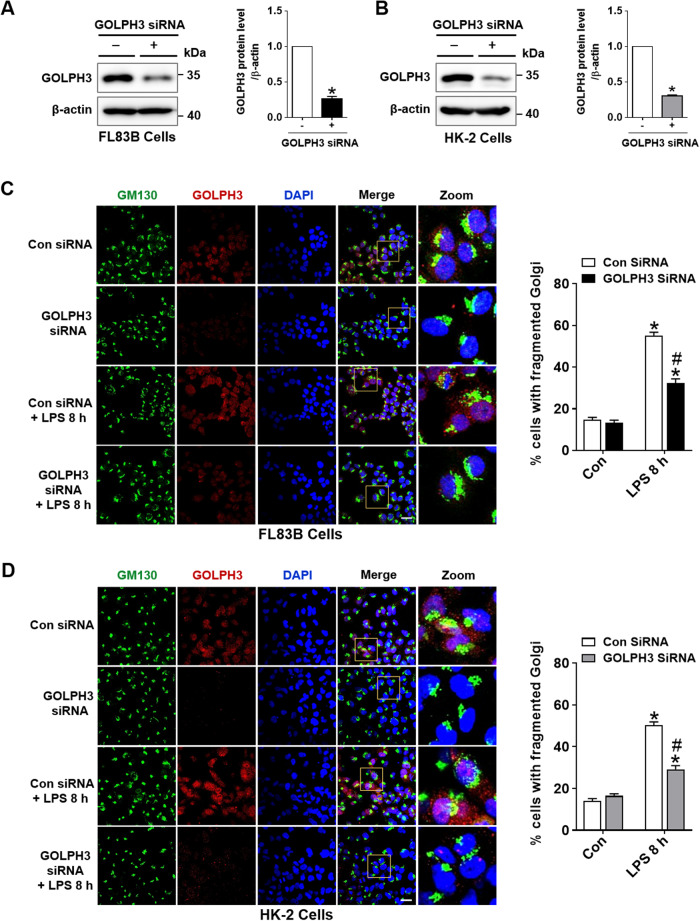
GOLPH3 knockdown decreases Golgi stress and fragmentation. FL83B and HK-2 cells were transfected with Con siRNA or GOLPH3 siRNA for 24 h, and then treated with LPS (1 µg/ml) for 8 h. **A**, **B** Western blot analysis was performed to evaluate transfection efficiency of GOLPH3 siRNA after 24 h. **C**, **D** After transfection and LPS treatment, the Golgi morphology of cells were examined. Representative images of immunofluorescence staining of GM130 (green) and GOLPH3 (red), and the quantification of cells with fragmented Golgi from each group are shown (*n* = 3). Scale bar, 20 µm. The data are presented as mean ± SEM. Two-tailed Student’s *t*-test (in **A**, **B**) and One-way ANOVA, followed by Bonferroni’s multiple comparisons (in **C**, **D**) were used, *p < 0.05 versus Con siRNA alone, and ^#^*p* < 0.05 vs LPS with Con siRNA.

To investigate the role of GOLPH3 on Golgi morphology and function in LPS-treated cells, GOLPH3 was downregulated by siRNA approach. Firstly, FL83B or HK-2 cells were transfected with GOLPH3 siRNA and the knockdown efficiency was evaluated by western blot analysis (Fig. 4A, B). After transfection, the cells were treated with LPS and determined the effect of GOLPH3 knockdown on the Golgi stress response. Immunofluorescence staining of GOLPH3 and GM130 revealed that LPS treatment increased GOLPH3 and Golgi fragmentation; however, downregulation of GOLPH3 improved the Golgi morphology and distribution and suppressed its fragmentation, indicating decreased Golgi stress (Fig. 4C, D). These results suggest that GOLPH3 directly mediates Golgi stress in hepatic and renal tubular cells.

### Downregulation of GOLPH3 reduces LPS-induced inflammatory response, apoptosis, and AKT/NF-kB signaling activation in hepatocytes

**Fig. 5:**
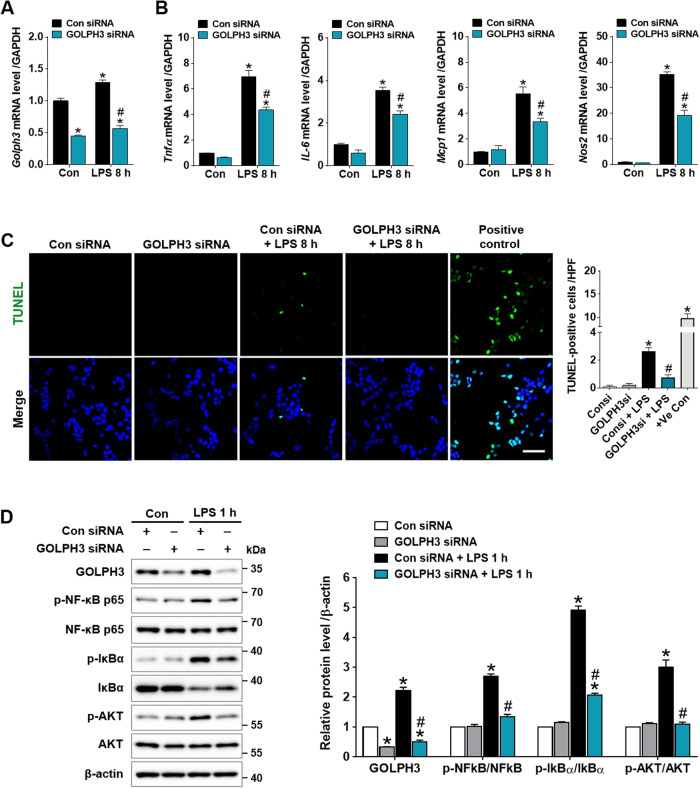
Downregulation of GOLPH3 reduces LPS-induced inflammatory response, apoptosis, and AKT/NF-κB signaling activation in hepatocytes. FL83B cells were transfected with Con siRNA or GOLPH3 siRNA for 24 h, and treated with LPS for 8 h. **A**, **B** After transfection, relative mRNA levels of *Golph3* and pro-inflammatory mediators (*Tnfα, IL-6, Mcp1*, and *Nos2*) were examined using real-time PCR analysis. Relative mRNA expression was normalized to that of GAPDH (*n* = 3). **C** Representative images of TUNEL staining and the quantification of TUNEL-positive cells/HPF using Image J software (NIH) are shown. Recombinant DNase I (5 U/ml) treated cells were used as experimental positive control (*n* = 3). Scale bar, 50 µm. **D** After transfection, the cells were treated with LPS for 1 h, and the protein expression levels of GOLPH3, p-NF-κB p65, p-IκBα, p-AKT, and β-actin (as a loading control) were analyzed by western blot analysis (*n* = 3). The data are presented as mean ± SEM. One-way ANOVA, followed by Bonferroni’s multiple comparisons was used, *p < 0.05 versus Con siRNA alone, and ^#^*p* < 0.05 vs LPS with Con siRNA.

To elucidate the role of GOLPH3 on inflammatory response and apoptosis in LPS-treated hepatocytes, FL83B cells were transfected with GOLPH3 siRNA and subsequently treated with LPS for 8 h. The results demonstrated that LPS treatment markedly increased the expression of *Golph3* and pro-inflammatory mediators (*Tnfα, IL-6, Mcp1*, and *Nos2*), which was downregulated by GOLPH3 siRNA (Fig. 5A, B). Apoptosis was assessed by TUNEL staining; LPS treatment significantly increased the number of TUNEL-positive cells, which was decreased by GOPH3 siRNA (Fig. 5C). In addition, the phosphorylation of NF-κB, IκBα, and AKT was examined in LPS-treated FL83B cells to reveal the signaling pathways associated with GOLPH3 using western blot analysis. The results demonstrated that LPS treatment induced the phosphorylation of AKT, IκBα, and NF-κB p65, while GOLPH3 knockdown significantly reduced the phosphorylation (Fig. 5D). These findings indicate that GOLPH3 directly mediates hepatic induction of pro-inflammatory cytokines and apoptosis through AKT/NF-kB signaling pathway.

### Downregulation of GOLPH3 reduces LPS-induced inflammatory response, apoptosis, and AKT/NF-kB signaling activation in renal tubular cells

**Fig. 6:**
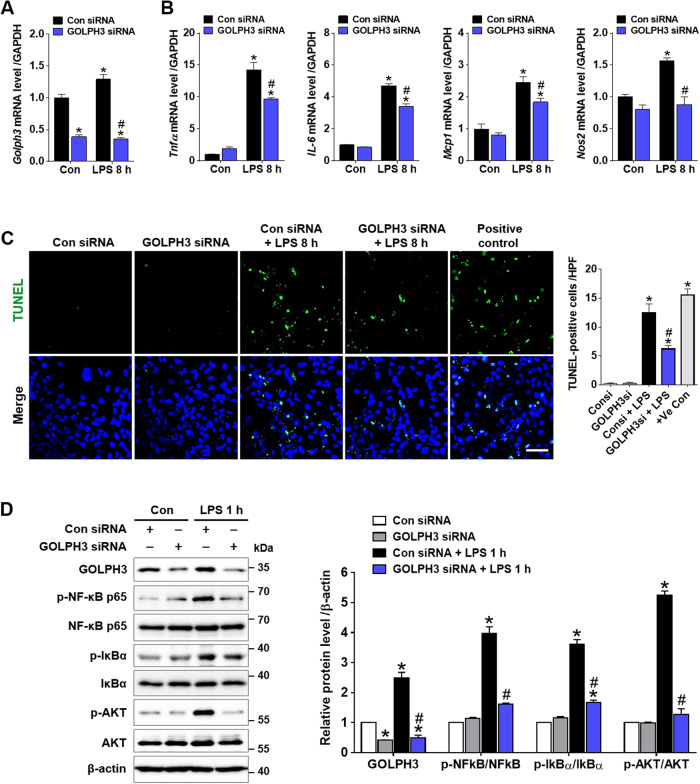
Downregulation of GOLPH3 decreases LPS-induced inflammatory response, apoptosis, and AKT/NF-kB signaling activation in renal proximal tubular cells. **A**, **B** After transfection of GOLPH3 siRNA for 24 h, the HK-2 cells were treated with LPS for 8 h, and examined relative mRNA levels of *Golph3* and pro-inflammatory mediators (*Tnfα, IL-6, Mcp1*, and *Nos2*) using real-time PCR analysis. Relative mRNA expression was normalized to that of GAPDH (*n* = 3). **C** Representative images of TUNEL staining and quantification of TUNEL-positive cells/HPF using Image J software (NIH) are shown. Recombinant DNase I (5 U/ml) treated cells were used as experimental positive control (*n* = 3). Scale bar, 50 µm. **D** After transfection, the cells were also treated with LPS for 1 h, and the protein expression levels of GOLPH3, p-NF-κB p65, p-IκBα, p-AKT, and β-actin (as a loading control) were analyzed by western blot analysis (*n* = 3). The data are presented as mean ± SEM. One-way ANOVA, followed by Bonferroni’s multiple comparisons was used, *p < 0.05 versus Con siRNA alone, and ^#^*p* < 0.05 vs LPS with Con siRNA.

To determine whether GOLPH3 downregulation decreases inflammation and apoptosis upon LPS treatment, HK-2 cells were transfected by GOLPH3 siRNA and treated with LPS for 8 h. The real-time PCR results showed that LPS increased the expression of *Golph3* and inflammatory mediators (*Tnfα, IL-6, Mcp1*, and *Nos2*). LPS treatment also increased TUNEL-positive apoptotic cells. Conversely, GOLPH3 knockdown significantly reduced the expression of *Golph3* and pro-inflammatory mediators, and number of apoptotic cells (Fig. 6A–C). Then, we further explored whether GOLPH3 knockdown affects the AKT/NF-κB signaling activation in LPS-treated cells, where the levels of p-AKT, p-IκBα, and p-NF-κB p65 were reduced by GOLPH3 knockdown, indicating suppressed AKT/NF-κB signaling (Fig. 6D). These results support that GOLPH3 directly regulates the inflammatory response, Golgi stress and associated apoptosis in renal tubular cells.

### Downregulation of GOLPH3 reduces LPS-induced inflammatory response and AKT/NF-kB signaling activation in macrophages

**Fig. 7:**
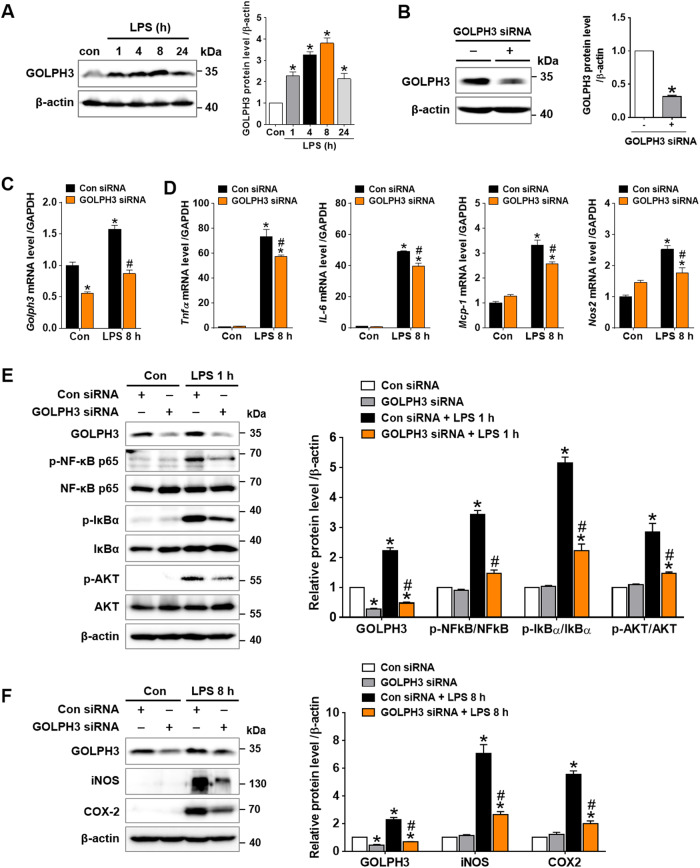
Downregulation of GOLPH3 suppresses LPS-induced inflammatory response and AKT/NF-κB signaling activation in macrophages. RAW 264.7 cells were treated with LPS (1 µg/ml) for different time points as indicated. **A** After LPS treatment, GOLPH3 expression was examined by western blot analysis (*n* = 3). **B** The cells were transfected with Con siRNA or GOLPH3 siRNA for 24 h and the knockdown efficiency was determined by western blot analysis. **C**, **D** The cells were transfected with Con siRNA or GOLPH3 siRNA for 24 h, and subsequently treated with LPS for 8 h. The relative mRNA levels of *Golph3* and pro-inflammatory mediators (*Tnfα, IL-6, Mcp1*, and *Nos2*) were determined using real-time PCR analysis. Relative mRNA expression was normalized to that of GAPDH (*n* = 3). **E** After transfection, the cells were treated with LPS for 1 h, and determined the protein expression levels of GOLPH3, p-NF-κB p65, p-IκBα, p-AKT, and β-actin (as a loading control) using western blot analysis (*n* = 3). **F** Cells were transfected with Con siRNA or GOLPH3 siRNA for 24 h and treated with LPS for 8 h, and cell lysates were analyzed by western blotting to measure GOLPH3, iNOS, and COX2 expression. The data are presented as mean ± SEM. One-way ANOVA, followed by Bonferroni’s multiple comparisons (in **A**, **C**, **D**, **E**, **F**) and two-tailed Student’s *t*-test (in **B**) were used, *p < 0.05 versus Con siRNA alone, and ^#^*p* < 0.05 vs LPS with Con siRNA.

Macrophages play important roles in cellular surveillance, immune response, tissue injury, and repair [42–44]. Hepatic or renal resident macrophages are major sources of pro-inflammatory cytokines and chemokines, which contribute to further tissue injury, inflammation, and subsequent fibrosis in various acute liver and kidney diseases [42–44]. First, we confirmed the induction of inflammatory cytokines in LPS-treated macrophages, RAW 264.7 cells (Supplementary data 2D). GOLPH3 was also upregulated after LPS treatment in a time-dependent manner (Fig. 7A, Supplementary data 2C). After transfection of GOLPH3 siRNA (Fig. 7B), the cells were treated with LPS and determined the expression levels of *Golph3* and inflammatory cytokines (*Tnfα, IL-6, Mcp1*, and *Nos2*) by real-time PCR analysis. GOLPH3 knockdown significantly decreased the levels of *Golph3* and these cytokines in LPS-treated cells (Fig. 7C, D). In LPS-activated macrophages [45], p-AKT, p-IκBα, and p-NF-κB p65 levels were upregulated, which was reduced by GOLPH3 knockdown, indicative of suppressed AKT/NF-κB signaling (Fig. 7E). We also investigated whether GOLPH3 pathway is associated with iNOS and COX-2 production, key players of inflammatory response in activated macrophages. LPS treatment significantly increased iNOS and COX-2 protein levels, which was reduced by GOLPH3 knockdown (Fig. 7F). These results suggest that GOLPH3 downregulation directly suppresses inflammatory response through AKT/NF-κB signaling activation in LPS-treated macrophages.

## Discussion

**Fig. 8:**
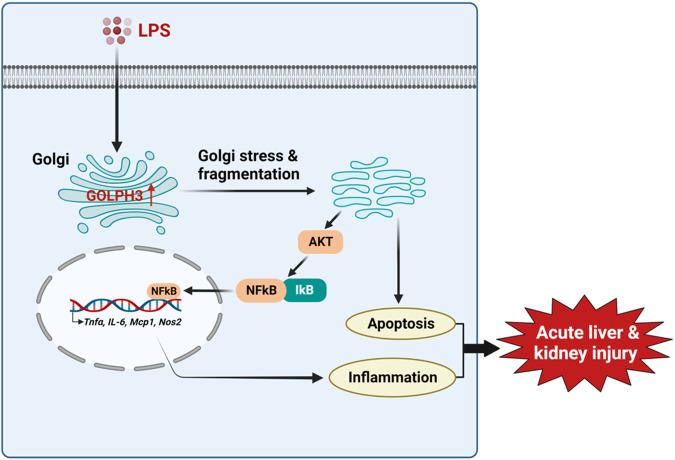
Proposed molecular mechanism of GOLPH3 in endotoxemia-induced acute liver and kidney injury.

In the present study, we showed that upregulation of GOLPH3 was strongly associated with Golgi stress and inflammatory response in the development and progression of acute liver and kidney injury. The results demonstrated that LPS-induced endotoxemia increased GOLPH3 expression, followed by the induction of Golgi stress and excessive pro-inflammatory mediators (*Tnfα, IL-6*, and *IL-1β)* in mice. Using in vitro LPS-treated hepatocytes and renal tubular cells, we consistently found that GOLPH3 is markedly increased, accompanied with the induction of Golgi stress and pro-inflammatory mediators (*Tnfα, IL-6*, and *IL-1β)*. Conversely, the downregulation of GOLPH3 significantly suppressed LPS-induced Golgi stress and inflammatory mediators (*Tnfα, IL-6, Mcp1,* and *Nos2)*, and reversing apoptotic cell death. In addition, the downregulation of GOLPH3 reduced inflammatory response in LPS-treated macrophages. Mechanistically, GOLPH3 knockdown significantly reduced inflammatory response and apoptosis by inhibiting AKT/NF-kB signaling activation and promoting the recovery of Golgi morphology and function. These findings highlight a critical role of GOLPH3 in regulating the development and progression of acute liver and kidney injury induced by endotoxemia. A proposed molecular mechanism of GOLPH3 is illustrated in Fig. 8.

Multiple organ dysfunction is a common feature found in the pathogenesis of PAMP- or DAMP-induced hyper-inflammatory processes in sepsis [7, 8]. The liver and kidney play important roles in many physiological and pathological processes, and these organs are much more vulnerable than others to circulating antigens, danger signals, and endotoxins following microbial infection [9, 10]. Therefore, recognition of liver and kidney dysfunction and its clinical significance in the course of sepsis development is a great importance for treatment. LPS is widely used to establish an experimental model of endotoxemia-induced organ injury [10, 15]. This study demonstrated that LPS increased pro-inflammatory cytokines in liver or kidney tissues (*Tnfα, IL-6*, and *IL-1β)*. The precise molecular mechanisms involved in the pathogenesis of liver and kidney injury are actively investigated.

The Golgi apparatus plays an important role in post-translational modification, trafficking and sorting of lipids and proteins synthesized from ER [16–18]. The proper Golgi organization plays important roles in the secretory and detoxifying function of the liver [16], and the Golgi apparatus is a common downstream effector of stress signals in cell death pathways [18, 19]. During apoptosis, secretory and trafficking pathways are blocked and cause the Golgi complex disassembly and fragmentation, resulting in Golgi stress [18]. The Golgi apparatus has been involved in the pathogenesis of diseases including neurological disorders, stroke, alcohol-related liver diseases, viral hepatitis, hepatocellular carcinoma (HCC), and renal cell carcinoma (RCC) [16, 18–23, 31]. This study presents that LPS-induced acute liver and kidney injury is associated with disruption of Golgi morphology evidenced by downregulation of Golgi reassembly-stacking protein (GRASP65) and an increase of regulatory proteins in the CREB3 and ARF4 pathway, resulting in Golgi stress response [41]. Molecular targets involved in Golgi stress response in the pathogenesis of liver or kidney injury are being actively investigated to support the discovery of new therapeutic strategies.

GOLPH3 is a Golgi membrane protein that regulates the maintenance of Golgi structure, secretory and trafficking pathways, and cellular stress signaling processes [18, 24]. GOLPH3 has been identified as an oncogenic protein, because it causes Golgi dispersal and fragmentation in response to DNA damage [25], and is frequently upregulated in many solid human tumors including HCC and RCC [27, 28, 46]. This suggests that GOLPH3 may have a critical role in hepatic and renal abnormalities. In this study, LPS-injected mice significantly upregulated GOLPH3 protein and mRNA expression in the liver and kidney, which was strongly correlated with endotoxemia-induced liver and kidney dysfunction. Consistently, GOLPH3 was increased in hepatic and renal cells in vitro after LPS treatment. Importantly, downregulation of GOLPH3 attenuated Golgi stress and inflammatory responses in an in vitro model of endotoxemia, suggesting a clear association of GOLPH3 with endotoxemia-induced liver and kidney dysfunction.

Under cytotoxic or stress conditions, disruption of Golgi morphology and function alters cellular redox balance and survival mechanisms, which aggravates excessive oxidative stress and cell death, contributing to the pathogenesis of many human disorders, including brain ischemic injury, endotoxemia-induced lung injury, and diabetic neuroinflammation [20, 21, 26, 31, 47]. A recent study showed that GOLPH3 is upregulated in neuroblastoma N2A cells upon oxygen-glucose deprivation and reoxygenation to promote stress-related autophagy, ROS production, and apoptotic cell death [30]. In endotoxemia-induced acute lung injury, GOLPH3 is highly increased and associated with oxidative stress, inflammation, and apoptosis; which were alleviated by activation of heme oxygenase-1 through reduction of Golgi fragmentation and GOLPH3 expression [31]. Hydrogen sulfide treatment reduces Golgi stress-induced apoptotic cell death and oxidative stress and ameliorates skeletal muscle atrophy in mice [47]. Accordingly, accumulating evidence suggests that GOLPH3 acts as a sensor of cellular stress and propagates Golgi stress to the downstream signaling effectors. Here, we investigated the role of GOLPH3 on LPS-mediated apoptosis in hepatocytes and renal tubular cells. The results showed that downregulation of GOLPH3 reduced Golgi fragmentation and apoptotic cell death, suggesting that GOLPH3 has a critical role to induce Golgi stress-related apoptosis in hepatocytes and renal cells.

The transcription factor NF-κB regulates inflammatory responses in many cell types including tissue-resident macrophages, Kupffer cells and kidney resident cells [8, 48, 49]. Activation of NF-κB leads to the transcription of pro-inflammatory genes encoding cytokines and chemokines and regulates both innate and adaptive immune responses [8, 48, 49]. The binding of IκBα protein to NF-κB masks its nuclear localization and effectively sequesters NF-κB in the cytoplasm. The PI3K/AKT has been identified as an upstream kinase for NF-κB activation, and acts indirectly by phosphorylating IκBα, which in turn dissociates IκBα from the NF-κB complex, leading to the transcriptional activation of inflammatory mediators [50, 51]. Pharmacological inhibition of NF-κB activity downregulates inflammatory response and related cell death signaling in many diseases including acute liver and kidney injury [8, 48, 51]. Furthermore, a recent study reported that GOLPH3 promotes angiogenesis and cancer cell proliferation by activating NF-κB signaling pathway [28]. This study investigated the effect of GOLPH3 on NF-κB activation in the endotoxemia-induced liver and kidney injury. GOLPH3 knockdown significantly decreased AKT and IκBα phosphorylation, simultaneously suppressing NF-κB activation and resulting in a reduced expression of pro-inflammatory mediators (*Tnfα, IL-6, Mcp1,* and *Nos2*) in LPS-treated hepatocytes and renal tubular cells as well as macrophages. Interestingly, GOLPH3 knockdown markedly reversed an increase in iNOS and COX-2 protein expression. These findings suggest that GOLPH3 plays an important role in the regulation of endotoxemia-induced inflammatory response through AKT/NF-κB signaling pathway. There are some limitations in the current study. First, we did not study the role of GOLPH3 on the endotoxemia-induced mouse model by manipulating its gene expression; further studies may help elucidating the effect of GOLPH3 in vivo. Second, tissue-specific functions of GOLPH3 may be helpful for developing target-oriented therapeutic strategies.

In summary, we demonstrated that GOLPH3 promotes acute liver and kidney injury induced by endotoxemia, associated with Golgi stress, inflammatory responses, and apoptotic cell death. Downregulation of GOLPH3 ameliorates the Golgi stress response through inhibition of AKT/NF-κB signaling and suppresses inflammatory response and apoptotic cell death. In conclusion, GOLPH3 is a novel therapeutic target in endotoxemia-induced acute liver and kidney injury by regulating the Golgi stress response.

## Supplementary information

Supplementary materials

Reproducibility checklist

Original Data File

## Data Availability

All data generated or analyzed during this study are available from the corresponding author on reasonable request.
