# Supplementary material for: GOLPH3 promotes endotoxemia-induced liver and kidney injury through Golgi stress-mediated apoptosis and inflammatory response
Source: Cell Death Dis. 2023 Jul 21;14(7):458. doi: 10.1038/s41419-023-05975-x (PMC10361983; doi:10.1038/s41419-023-05975-x)
Supplement: Supplementary file 1 — Supplementary materials [file 41419_2023_5975_MOESM1_ESM.docx]

**Supplementary Table 1. Antibodies used in this study**

| **Antibody** | **Vendor** | **Catalog No.** | **Host** | **Application** |
| --- | --- | --- | --- | --- |
| GOLPH3 | Proteintech | #19112-1-AP | Rabbit | 1:2000 (WB)  3 µg/ml (IHC)  3 µg/ml (IF) |
| GRASP65 | Proteintech | #10747-2-AP | Rabbit | 1:1000 (WB) |
| ARF4 | Proteintech | #11673-1-AP | Rabbit | 1:1000 (WB) |
| PI4KIIIβ | Abcam | #ab109419 | Rabbit | 1:1000 (WB) |
| AKT | Cell Signaling | #9272 | Rabbit | 1:1000 (WB) |
| p-AKT^Ser473^ | Cell Signaling | #9271 | Rabbit | 1:500 (WB) |
| IkBα | Cell Signaling | #9242 | Rabbit | 1:1000 (WB) |
| p-IkBα^Ser32^ | Cell Signaling | #2859 | Rabbit | 1:500 (WB) |
| NF-kB p65 | Cell Signaling | #8242 | Rabbit | 1:1000 (WB) |
| p- NF-kB p65^Ser536^ | Cell Signaling | #3033 | Mouse | 1:500 (WB) |
| iNOS | Cell Signaling | #2982 | Rabbit | 1:1000 (WB) |
| COX-2 | Cell Signaling | #12282 | Rabbit | 1:1000 (WB) |
| MYO18A | Santa Cruz | #sc-365328 | Mouse | 1:1000 (WB) |
| Goat anti-rabbit HRP conjugate | Bio-rad | #170-6515 | Rabbit | 1:5000 (WB) |
| Goat anti-mouse HRP conjugate | Bio-rad | #170-6516 | Mouse | 1:5000 (WB) |
| β-actin | Sigma-Aldrich | #A5441-2ML | Mouse | 1:20,000 (WB) |
| GM130 | BD Biosciences | #610823 | Mouse | 2.5 µg/ml (IF) |
| Goat anti-mouse IgG-Alexa Fluor 488-Green | Abcam | #ab150113 | Mouse | 2 µg/ml (IF) |
| Goat anti-mouse IgG-Alexa Fluor 594-Red | Abcam | #ab150080 | Rabbit | 2 µg/ml (IF) |

**Supplementary Table 2. The primer sequences used for real-time PCR analysis in this study**

| **Gene** | | **Forward primers (5’-3’)** | **Reverse primers (3’-5’):** |
| --- | --- | --- | --- |
| Mouse | *Arf4* | CTGGAGGCATTACTTCCAGAATACC | TCCCTCATACAGACCAGTTCCTTG |
|  | *Creb3* | GTGAAGGCTCCGCTGGACTTAG | AGATCTATGGAGACGTGCTCCTGT |
|  | *Golph3* | AGCAGCGTCTCATCAAGAAG | AGGCATGGGCTAGGTAAATG |
|  | *Grasp65* | GCTGAAGGCCAATGTGGAGAAG | CACATCCAGCACATGCCACAC |
|  | *IL-1β* | TCGCAGCAGCACATCAACAAGAG | GGTGCTCATGTCCTCATCCTGGA |
|  | *IL-6* | GACTTCCATCCAGTTGCCTTCTTG | GGTATCCTCTGTGAAGTCTCCTCT |
|  | *Mcp1* | ACCTTTGAATGTGAAGTTGA | CTACAGAAGTGCTTGAGGTG |
|  | *Myo18a* | CTGTAGCCCAGACTGCATATAG | AGATGCTGAAAGCTGGTAGTC |
|  | *Nos2* | GGAATCTTGGAGCGAGTTGT | CCTCTTGTCTTTGACCCAGTAG |
|  | *Tnfα* | CATATACCTGGGAGGAGTCT | GAGCAATGACTCCAAAGTAG |
|  | *Gapdh* | GTGGCAAAGTGGAGATTGTTG | TTGACTGTGCCGTTGAATTTG |
| Human | *Golph3* | AGGTTACAACTAGAGGCTTGTGG | CCTGTTGGAGCATCTGACTTACAG |
|  | *IL-1β* | CAAAGGCGGCCAGGATATAA | CTAGGGATTGAGTCCACATTCAG |
|  | *IL-6* | CCAGGAGAAGATTCCAAAGATGTA | CGTCGAGGATGTACCGAATTT |
|  | *Mcp1* | GGCTGAGACTAACCCAGAAAC | GAATGAAGGTGGCTGCTATGA |
|  | *Nos2* | CAGTGACACAGGATGACCTTCAGT | GTTGCATCCAGCTTGACCAGAGA |
|  | *Tnfα* | TGCTGCAGGACTTGAGAAGA | GGCTACATGG-GAACAGCCTA |
|  | *Gapdh* | GGTGTGAACCATGAGAAGTATGA | GAGTCCTTCCACGATACCAAAG |


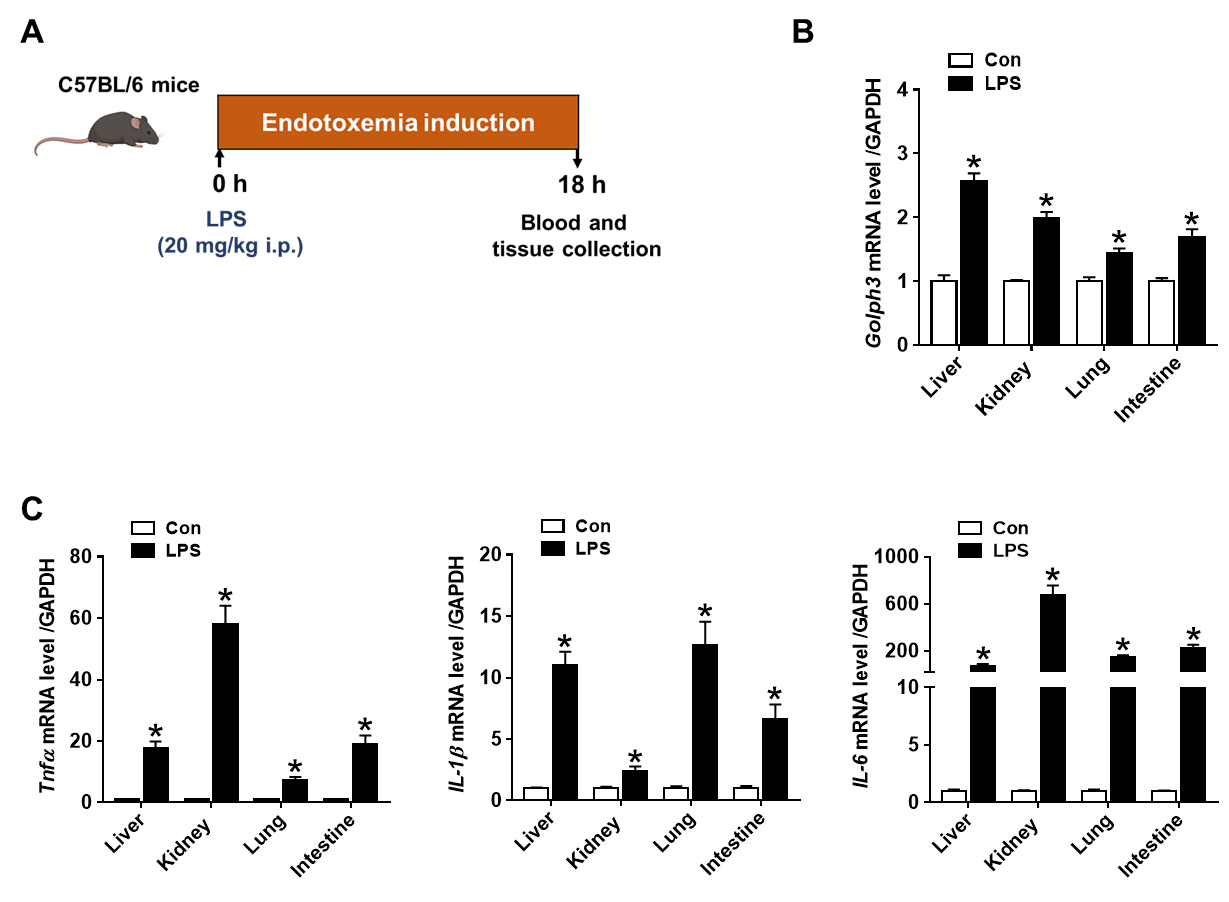


**Supplementary data 1. GOLPH3 mRNA expression in endotoxemia-induced acute liver and kidney injury in mice.** Endotoxemia-induced acute liver and kidney injury was induced in C57BL/6 mice by an intraperitoneal injection with a single dose of LPS (20 mg/kg) or normal saline (control), and the mice were sacrificed at 18 hours post-injection (A). Relative mRNA expression of *Golph3* and pro-inflammatory cytokines (*Tnfα, IL-1β,* and *IL-6*) was examined using real-time PCR analysis in various organs (liver, kidney, lung, and intestine); each gene expression was normalized to that of GAPDH (n = 3-5) (B, C). The data are presented as mean ± SEM. Two-tailed Student’s *t*-test was used, *p <0.05 versus control.


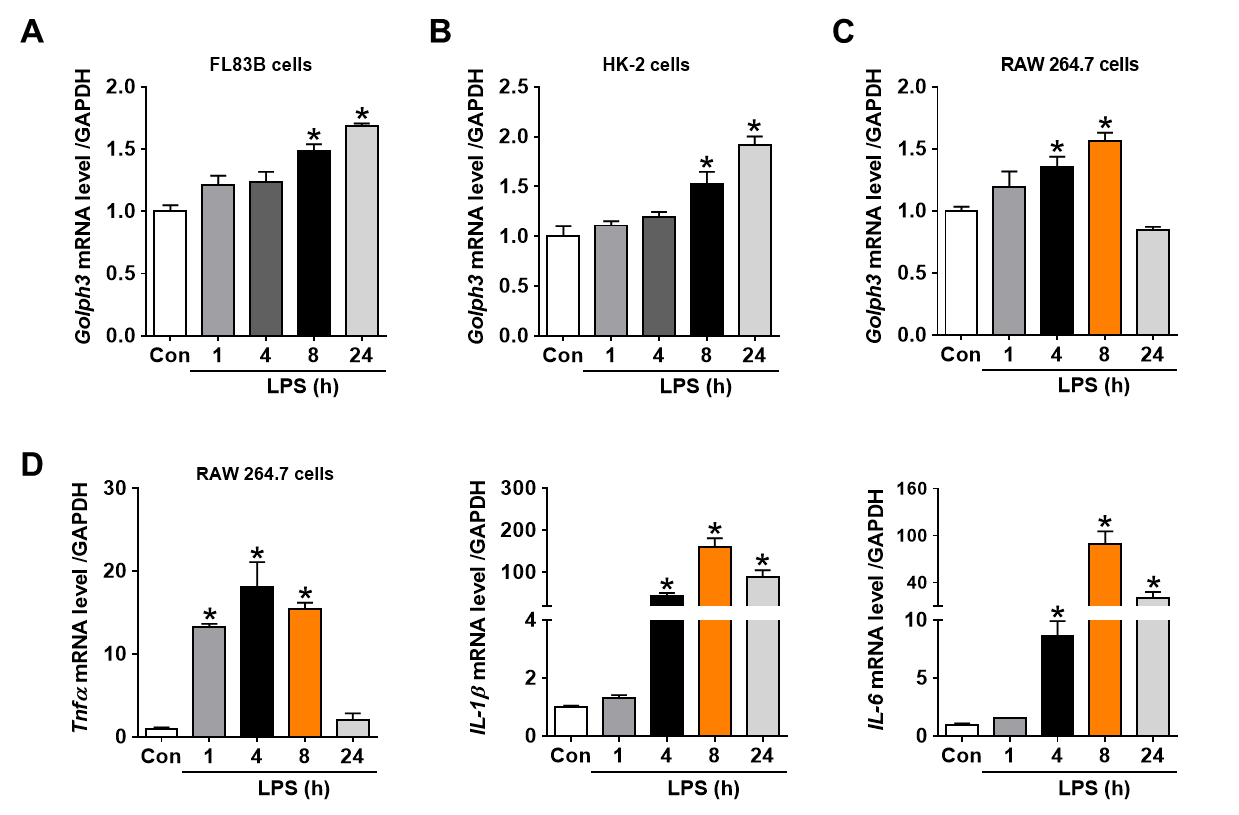


**Supplementary data 2. LPS treatment induces GOLPH3 and inflammatory cytokine expression *in vitro.*** The cells were treated with LPS (1 µg/ml) in time-dependent manner as indicated. The relative mRNA expression of *Golph3* in FL83B, HK-2, and RAW264.7 cells (A-C), and pro-inflammatory cytokines (*Tnfα, IL-1β,* and *IL-6*) in RAW264.7 cells (D) were respectively evaluated using real-time PCR analysis after LPS treatment. Each gene expression was normalized to that of GAPDH (n = 3). The data are presented as mean ± SEM. One-way ANOVA, followed by Bonferroni’s multiple comparisons were used, *p <0.05 versus control.
