# Supplementary material for: GOLPH3 promotes endotoxemia-induced liver and kidney injury through Golgi stress-mediated apoptosis and inflammatory response
Source: Cell Death Dis. 2023 Jul 21;14(7):458. doi: 10.1038/s41419-023-05975-x (PMC10361983; doi:10.1038/s41419-023-05975-x)
Supplement: Supplementary file 3 — Original Data File [file 41419_2023_5975_MOESM3_ESM.pdf]

Western blot_Original membranes

Figure 1E

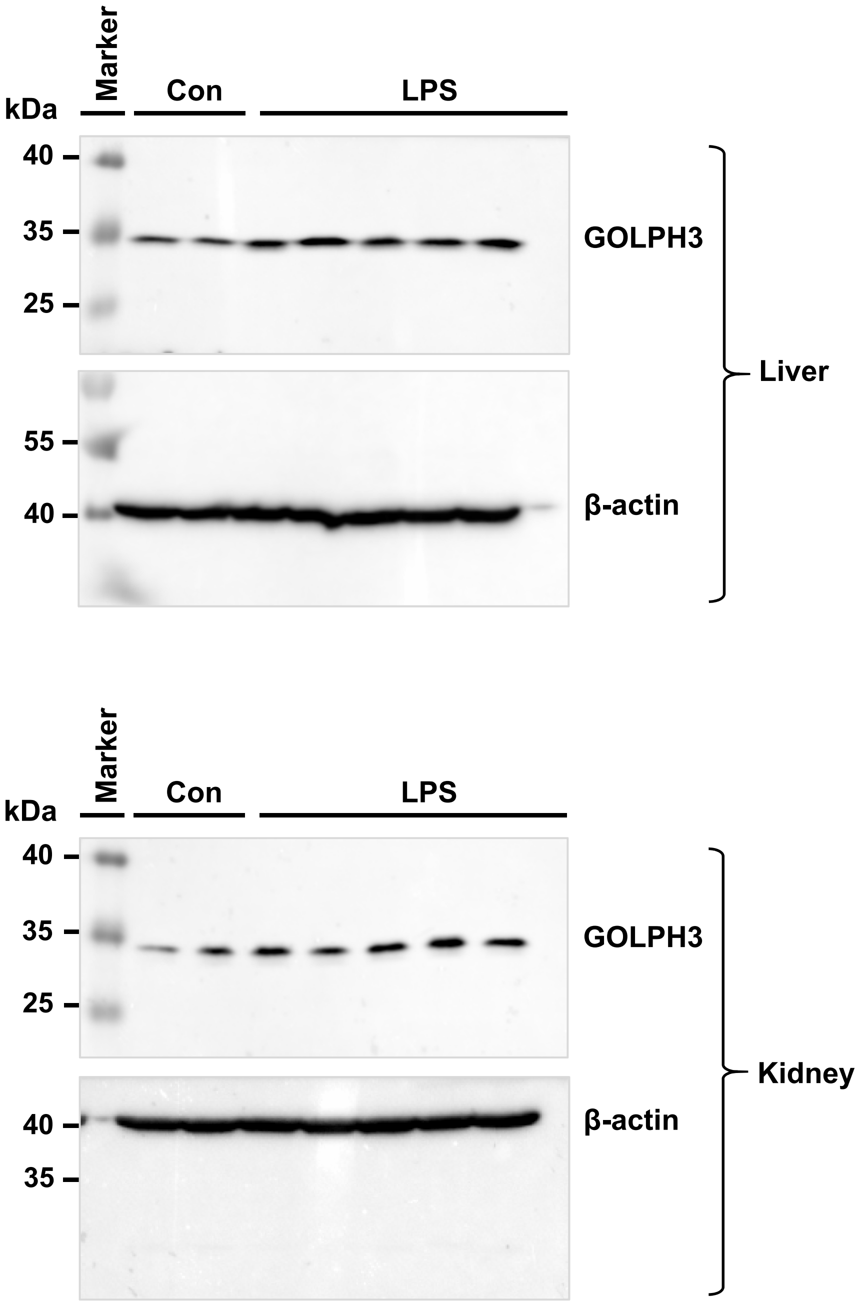

Figure 2B

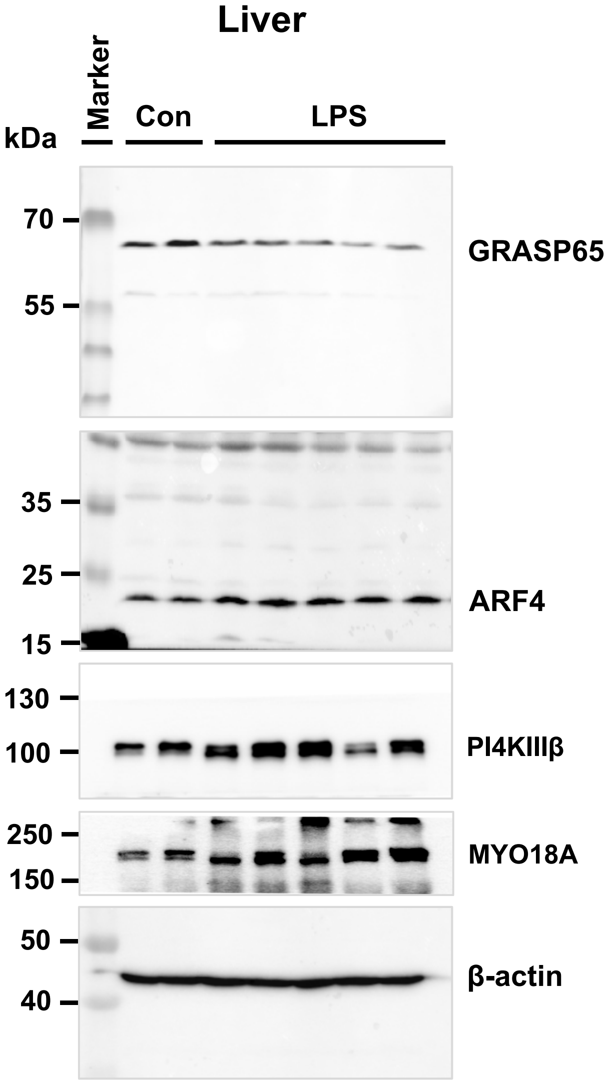

Figure 2H

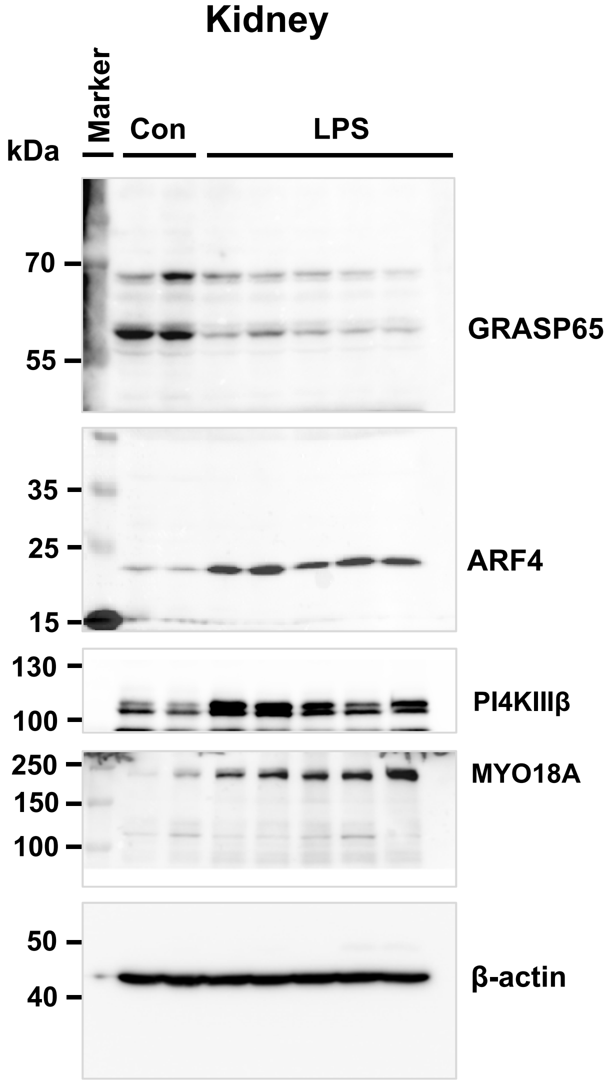

Figure 3A

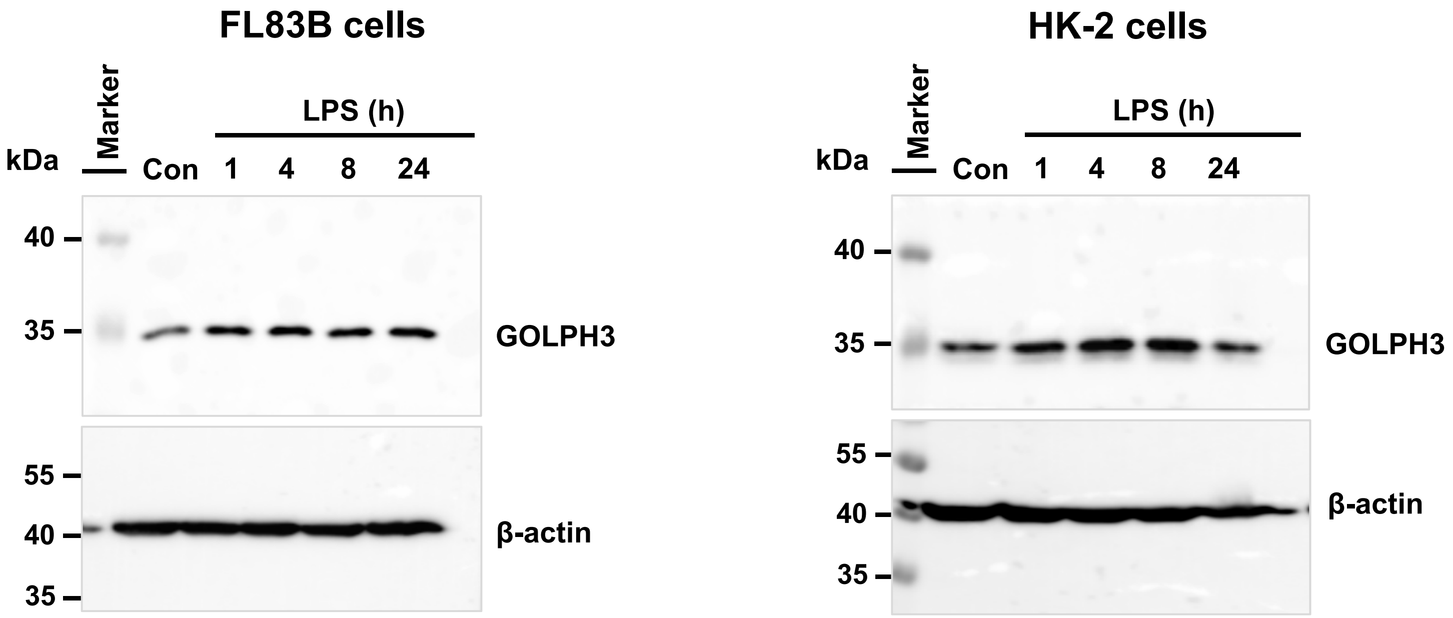

Figure 4A

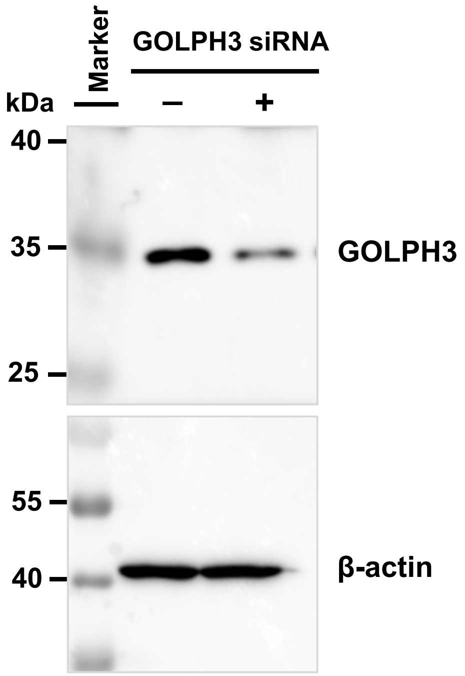

Figure 4B

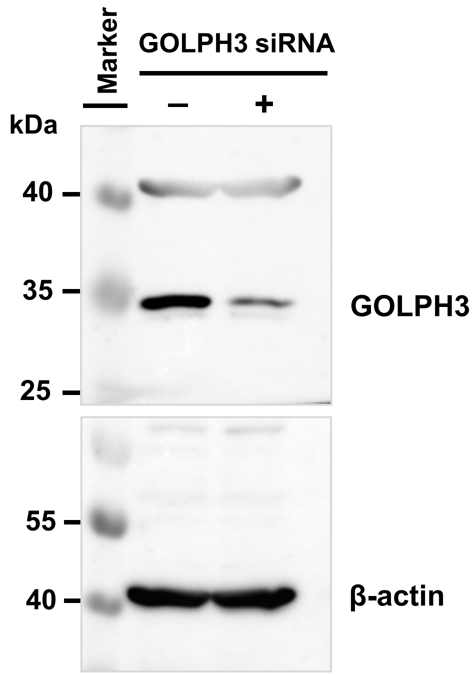

Figure 5A

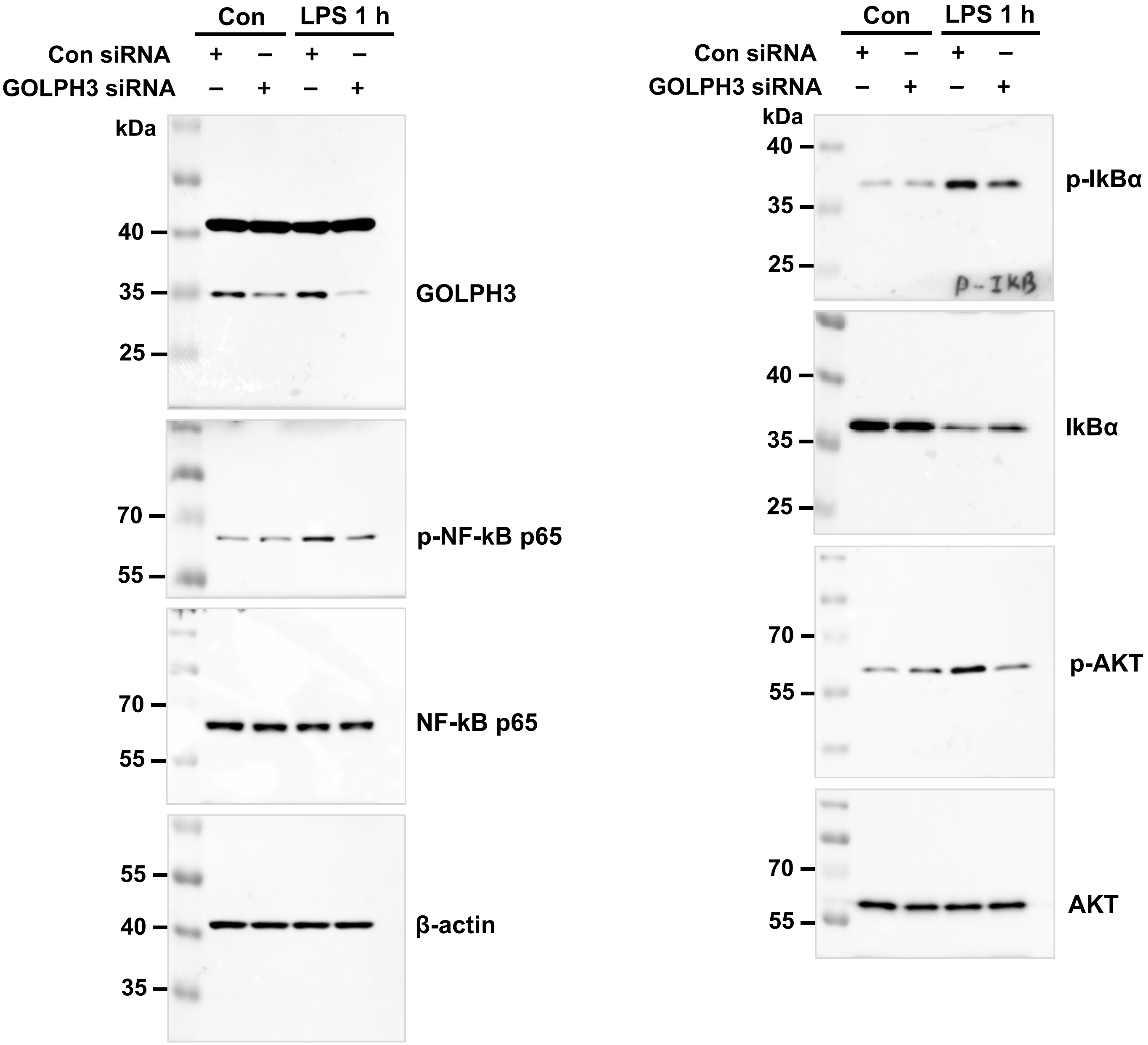

Con
LPS 1 h
Con siRNA + − + −
GOLPH3 siRNA − + − +
kDa
40
35
25
GOLPH3
70
55
p-NF-kB p65
70
55
NF-kB p65
55
40
35
β-actin
Con
LPS 1 h
Con siRNA + − + −
GOLPH3 siRNA − + − +
kDa
40
35
25
p-IkBα
40
35
25
IkBα
70
55
p-AKT
70
55
AKT

Figure 6A

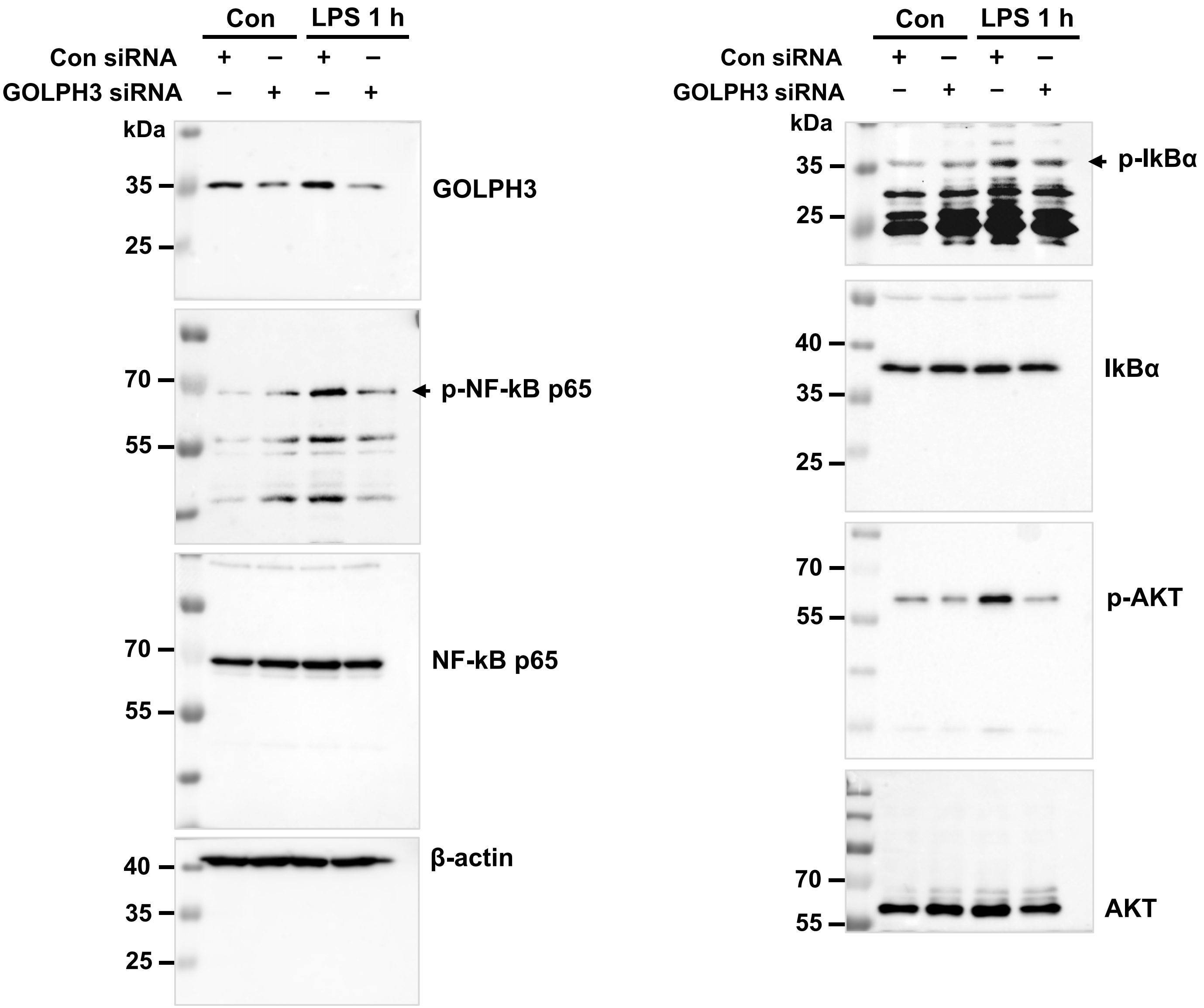
Con
LPS 1 h
Con siRNA + − + −
GOLPH3 siRNA − + − +
kDa
35
25
GOLPH3
70
55
p-NF-kB p65
70
55
NF-kB p65
40
35
25
β-actin
Con
LPS 1 h
Con siRNA + − + −
GOLPH3 siRNA − + − +
kDa
35
25
p-IkBα
40
35
25
IkBα
70
55
p-AKT
70
55
AKT

Figure 7A

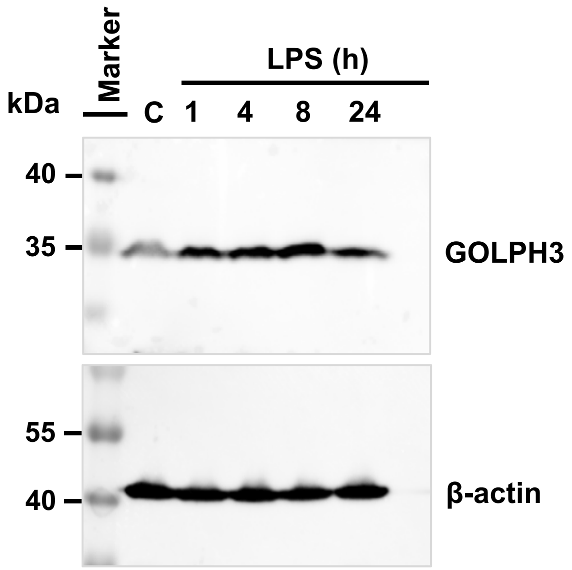

Figure 7B

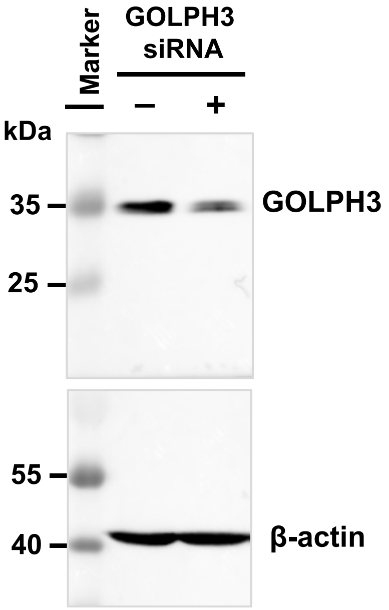

Figure 7E

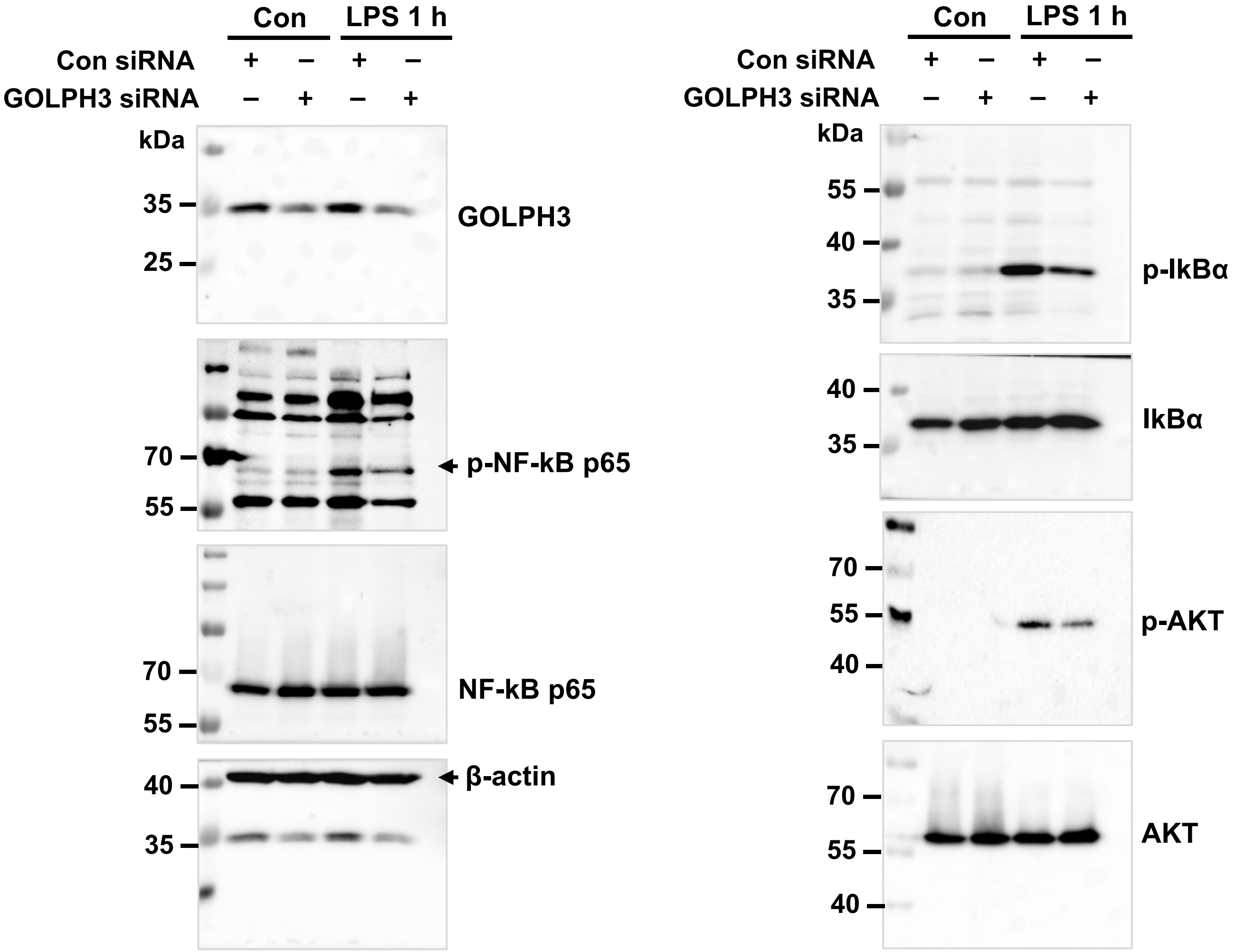

Figure 7F

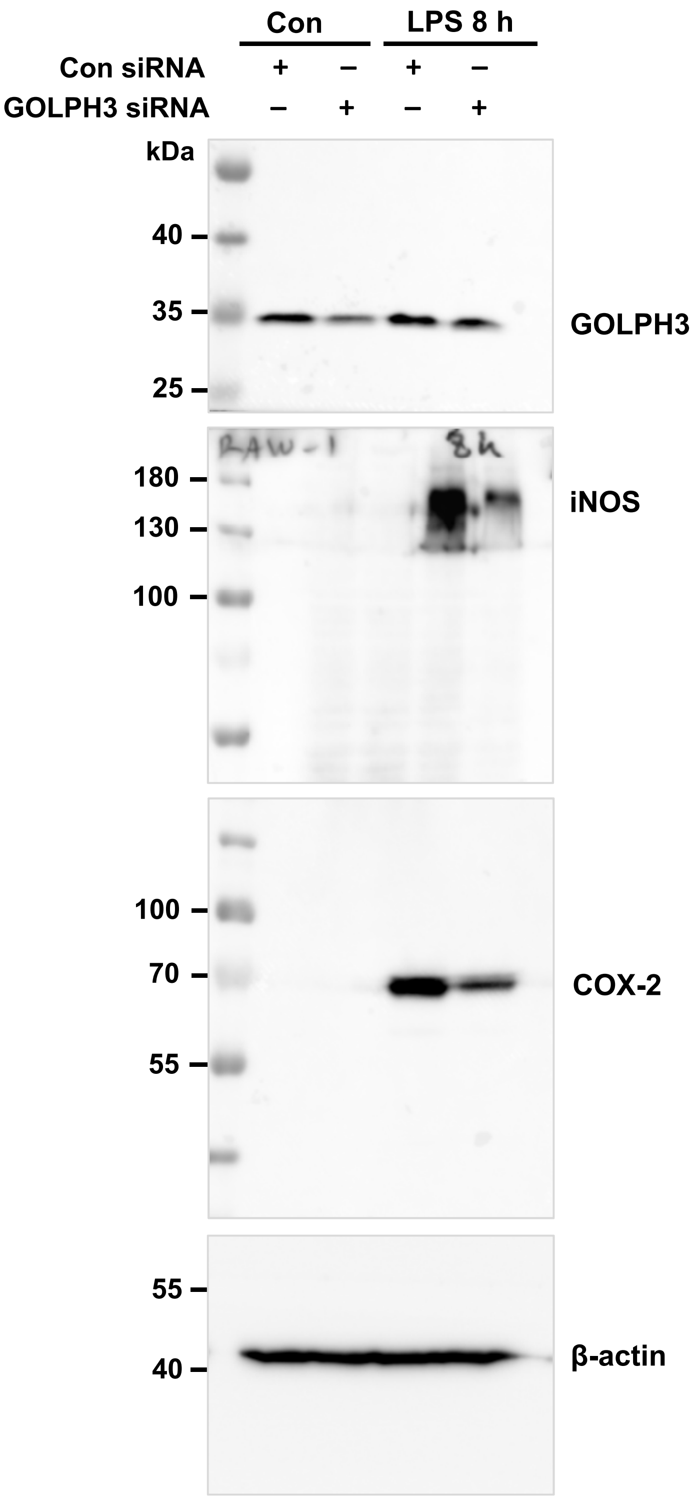

Con
LPS 8 h
Con siRNA + − + −
GOLPH3 siRNA − + − +
kDa
40
35
25
GOLPH3
RAW-1
8h
180
130
100
iNOS
100
70
55
COX-2
55
40
β-actin
